# Health literacy on non-communicable diseases prevention among adults in Northwest of Amhara region, Ethiopia: A qualitative study

**DOI:** 10.1371/journal.pone.0354160

**Published:** 2026-08-13

**Authors:** Nakachew Mekonnen Alamirew, Eshetu Girma, Sahai Burrowes, Yohannes Kebede Lemu

**Affiliations:** 1 Department of Health, Behavior, & Society, Faculty of Public Health, Jimma University, Jimma, Oromia, Ethiopia; 2 Department of Public Health, Debre Markos University, College of Health Sciences, Debre Markos, Ethiopia; 3 Research Scientist, African Population and Health Research Centre, Nairobi, Kenya; 4 Public Health Program, Touro University California, Vallejo, California, United States of America; Hamadan University of Medical Sciences, IRAN, ISLAMIC REPUBLIC OF

## Abstract

**Introduction:**

Health literacy is a multidimensional concept that encompasses a range of skills people need to function effectively and efficiently in a healthcare environment. This study, aims to fill the gap in knowledge about health literacy regarding noncommunicable disease prevention practices in the Ethiopian context.

**Methods:**

A descriptive qualitative study was conducted to investigate health literacy-oriented noncommunicable disease prevention behaviors in the Amhara Region of Northwest Ethiopia. The study used focus group discussions and in-depth interviews with a purposive sample of 51 diverse participants from the community and health system. To further understand participants’ health literacy on common noncommunicable diseases, a thematic analysis approach was employed. Atlas TI. 9.1.3.0 software was used for data analysis.

**Results:**

Four themes emerged regarding how health literacy relates to the prevention and control of noncommunicable diseases. Factors contributing to low level of preventive behavior for noncommunicable diseases include the level of health literacy, lack of isolated comprehensive preventive health services room, a carefree attitude towards health, operational failures, knowledge gaps, and motivational variables.

**Conclusion:**

Participants demonstrated inadequate health literacy and held misconceptions about non-communicable diseases and their associated risk factors. To reduce the growing burden of noncommunicable diseases, this study highlights the need to address the diverse health literacy needs of adults-including the knowledge and skills required to recognize and act on these conditions or behaviors, and the ability to understand and respond to health requirements by implementing context specific health literacy improvement strategies for noncommunicable disease literacy.

## Introduction

Health literacy (HL) is a multidimensional concept that addresses a range of skills people need to effectively and efficiently function in a healthcare environment [1,2]. It is developed over time through social practices and Sorensen et al. amended the definition in 2012 after conducting a comprehensive analysis of academic literature on HL definitions, identifying 17 variations that deviated from the fundamental concept that it is a synthesis of competencies that: *“*HL is linked to literacy and entails people’s knowledge, motivation and competence to access, understand, appraise and apply health information in order make judgments and take decisions in everyday life concerning health care, disease prevention, and health promotion to maintain to improve quality of life during the life course.” This definition included “The public health perspective”, which may be readily modified to support an individual approach by replacing the three domains of health—healthcare, disease prevention, and health promotion—with “being ill, being at risk, and staying healthy.” [3]. HL is now a multifaceted notion that encompasses social and cultural contexts, rather than just reading and numeracy skills as it was in the past [4–6].

HL plays a crucial role in the prevention and management of NCDs by empowering individuals to make informed decisions about their health behaviors and access to care. There has been a recent shift toward the understanding that HL is about the relationship between the skills of persons receiving care or treatment and systems that are providing the care and treatment [7]. It is essential for people to understand what NCDs risk factors and determinants are, whether or not they are at risk, the nature of the disease, and the daily health-related decisions they may need to make for the prevention and control of diseases for themselves, their families and their communities [8]. As well it is an important factor in disease prevention and control [9,10].

Evidence suggests that low HL is associated with adverse health outcomes [11,12], including frequent use of emergency care [11,13], prolonged hospital stays, and high mortality rates, which in turn lead to health disparities [13], and associated with increased health care costs [14]. Also, low HL is associated with decreased use of preventive services, poorer ability to interpret labels and health messages, poorer health status, higher mortality, and higher healthcare costs [11]. It also negatively impacts disease self-management and individual health behaviors such as adherence to weight control, tobacco cessation interventions and cancer screening recommendations [15]. Individuals with low HL are more likely to present with advanced illness, resulting in delayed diagnosis and treatment and poorer outcomes [16]. Yet, many individuals are unable to comprehend or act upon health information because of limited health literacy [17].

Despite the burden of NCDs in LMICs, as evidenced by [18], even if Ethiopia, prepared a National NCD targets and developed a National integrated NCD policy/strategy/action plan and Guidelines for management of cancer, CVD, diabetes and CRD, as per the WHO Global Strategy, still the Country has not implemented fully the ten NCD progress monitoring indicators, (a national public awareness and motivational communication for physical activity, unhealthy diet reduction measures, and Drug therapy/counselling to prevent heart attacks and strokes and partially implemented harmful use of alcohol reduction measures) at all as appropriate [19]. Globally and in developing nations like Ethiopia, despite its significance in self-care and the prevention of chronic diseases, HL is not well operationalized [20,21] and there is a lack of comprehensive qualitative studies, especially at community level. Public awareness efforts, population-level HL studies, and national NCD policies are lacking in Ethiopia. Tracking health literacy-oriented qualitative study is a key set of tracer actions link to interventions allows for national benchmarking and monitoring of progress being made against NCDs. It also serves to highlight challenges and areas requiring further attention. This study, the first of its kind in the region, fills the void by exploring HL on NCDs prevention practices in the specific context.

## Methods and materials

### Study design and setting

A descriptive qualitative study approach was Used. The study was conducted in semi-urban town communities located in the Amhara Region, Northwestern Ethiopia. The region is divided into 22 administrative zones. From these administrative zones, East Gojjam administrates Zones was purposively chosen. Two town administrator sites, representing 25% of the district town administrators in the zone, were selected for the study. The two town administrations were purposively selected based on their administrative structure, population size, and functional similarity to other small- and medium-sized towns in Ethiopia. These towns have comparable public health facilities, population structures, to capture variation in population characteristics, including differences in education level, economic status, access to health information sources, logistical feasibility, and established primary healthcare units offering NCD screening and health education services. This diversity allowed for a richer understanding of how HL influences NCD preventive behavior across different social groups and they maintain accessible health facilities that routinely interact with adult populations. These characteristics made the sites appropriate for examining how HL influences preventive behaviors among adults.

Health literacy development is undertaken across all settings where people’s knowledge, understanding, and behavior about health can be influenced – that is, all places where people are exposed to health-related information and where their health behaviors may be influenced [8]. In the region including the selected semi-urban town administrators there is a workflow strategy from the region to the health care setting to manage NCDs. The community is characterized by strong cultural traditions, close-knit social networks, and a high level of reliance on cultural and traditional norms as well as on local health workers for primary care services. In these settings, several community health volunteers play a vital role in health education and outreach activities. As this context represents a setting where resource constraints, cultural beliefs, and healthcare practices intersect in complex ways, understanding the local context was essential for interpreting participants’ perspectives and capturing the nuances of their lived experiences.

### Study population and sampling section

It has previously been recommended that qualitative studies require a minimum sample size of at least 12 to reach data saturation [22,23]. Therefore, a total sample of four focus group discussions (FGDs), each comprising 7–10 participants, and 18 in-depth interviews (IDIs) were conducted with community dwellers, individuals working at Kebele level who support the health extension program, and health extension workers (HEWs). The sample size was determined based on the principle of data saturation, whereby data collection continued until no new themes or insights emerged from successive interviews [24]. Criterion based purposive sampling based on predefined inclusion criteria (including: individual role, years of experience, residents of the selected semi-urban towns (living in a selected town administrations) individuals experienced with NCD (but not diseased), aged above 18 years, or involvement in the relevant service) was used to select participants and was deemed sufficient for the qualitative analysis and scale of this study. Individuals who did not meet these criteria or were unable to provide informed consent were excluded. This approach is consistent with qualitative research methods aimed at achieving depth and contextual understanding relevant to the study objectives rather than representativeness. The study was employed between May 12, 2024, and July 7, 2024.

### Data collection tools and procedure

The methodological orientation and theory underpinning this study were derived from the conceptual model of the European Health Literacy Survey (HLSEU) project [25]. Based on the HLS-EU conceptual model, the research team (NM, YK, EG and SB) developed semi-structured FGD and IDI guides. The conceptual model focuses on how people access, understand, appraise and apply information to make decisions in everyday life regarding healthcare, disease prevention and health promotion. It also demonstrates how HL is influenced by personal, situational and societal determinants. The model indicates how HL affects health service use and costs, health behavior and health status, social participation and empowerment, as well as equity and sustainability [25]. The study used FGDs and IDIs as data collection methods. The FGDs were facilitated by a team of experts, with the Principal Investigator (PI) (NM) serving as the discussion facilitator.

All IDIs were also conducted by the PI. Both the FGD and IDI guides included questions related to local customs and conditions relevant to the study setting, addressing two broad research questions: 1) What effect do different levels of HL have on people’s understanding, attitudes, and engagement in NCD prevention behaviors? and 2) What barriers and facilitators do people with inadequate HL face when accessing and using information on NCD prevention among adults? After each FGD, the facilitator (PI) and the two professionals who participated in each FGD made notes and discussed preliminary observations. Each FGD lasted approximately one hour, and the IDIs lasted a minimum of 30 minutes. A face-to-face method was used for both FGDs and IDIs, which took place in locations convenient for the participants. As recommended by Russell et al., (2008), the town health extension workers served as a formal community gatekeepers [26]. The researchers contacted the health centers (part of the primary health care unit in the tiered system of the country) and health extension workers for participant recruitment and obtained consent for voluntary participation. All participants completed the qualitative face-to-face interviews and FGD sessions in their native Amharic language.

### Study procedures

The data was coded by the PI as an independent coder (NM) and validated by YK. The transcripts (quotations, sentences or words) were labeled with codes to catalog key concepts while preserving the context in which these concepts occurred. The codes were then inductively clustered into initial concepts, categories, sub-themes, and themes which were compared and discussed to provide an overview of trends in views regarding health literacy in the study setting as expressed by the participants. Participants were asked to define and describe study-related constructs in their own words and conceptualizations as they relate to healthy living and were then guided through in-depth explorations of relevant influences. They were encouraged to elaborate on individual stories and collective narratives when discussing their perspectives and related issues and challenges. This approach allowed for less restrictive and richer responses from participants [27]. Prior to data collection, specialists examined the suitability and clarity of both interview and FGD protocols (YK, EG, SB). Before the FGDs and IDIs began, participants received an orientation about the study’s objectives and their responsibilities to minimize unwanted interruptions and protect their rights. Other specialists translated the Amharic transcription to English to ensure data uniformity and eliminate the lead investigator’s bias. Each IDIs and FGDs were recorded with a digital audio recorder. As much as feasible, recordings were made in tranquil environments.

### Data analysis

To gains a greater understanding of participants’ HL and perspectives to common NCDs specifically related to healthy behaviors and subsequently any adherence strategies they adopt for preventive action, thematic analysis was conducted following Braun and Clarke’s six-step framework [28], using a primarily inductive approach. Atlas Ti 9.1.3.0 was used to organize and manage data. Themes were developed iteratively and reviewed in relation to the research questions. As depicted in Table in S1 Table in S1 File; this report approaching a Consolidated criteria for reporting qualitative research (COREQ) [29].

### Rigor

In qualitative research, rigor refers to the study’s trustworthiness and quality, which can be ensured using several critical criteria. In this study, credibility was achieved by ensuring that the findings were accurate and convincing. Strategies such as triangulation (for a deeper understanding of the practice), the use of multiple data sources, and mixed data gathering techniques including IDIs, FGDs, and observation (the primary data collection method) were employed, and notes were collected accordingly. The transcribed data, interpretations, and conclusions were returned to a few participants for review to correct errors and challenge any potentially incorrect interpretations (member checking). One MPH professional at Debre Markos University reviewed the transcribed data to explore features of the inquiry and to uncover biases through peer debriefing. Extended involvement was ensured by observing participants for body language, including facial expressions, eye gaze, and tone of voice. Similarly, to enhance transferability the PI provided extensive, thorough descriptions to allow others to determine whether the findings apply to diverse contexts. To maintain consistency throughout the research process (dependability), records were preserved for an audit trail, and specific methodological principles were employed throughout the study. Additionally, to assure confirmability – that the findings reflect participant perspectives rather than researcher bias- reflexivity and open documentation were employed. Finally, to fairly represent different points of view (authenticity) participants’ views were heard and valued throughout the research process.

### Ethical consideration

This qualitative, cross-sectional, community-based study involved human participants and received ethical approval from the Institutional Review Board (IRB) of Jimma University (Ref. No. JUIH/IRB/050/24) and support letter was obtained from East Gojjam Zonal Health Department. The study was conducted in accordance with the ethical principles of the Declaration of Helsinki. Prior to participation, all individuals were provided with a clear explanation of the study objectives, qualitative data collection procedures (including interviews and/or focus group discussions), potential risks and benefits, measures to ensure confidentiality, and the voluntary nature of participation. Verbal informed consent was obtained from all participants prior to data collection and was audio-recorded. The use and recording of verbal consent were approved by the IRB, considering the community-based setting, cultural appropriateness, and the minimal risk posed to participants. Participant privacy was safeguarded through the collection of anonymized data, secure storage of all study materials, and restricted access to the data limited to the research team.

## Results

The study is based on four focus groups size varied from 7 to 10 participants and 18 IDIs with a total of 51 participants. As depicted in Table 1, of the participants, twenty (39.2%) were men. The median age for all participants was 30 years (minimum of 20 and maximum of 70 years). Approximately 1/4^th^ (25 percentile) of the participants’ is below the age of 27 and earn a monthly income of less than 1567.00 Ethiopian Birr with a median average monthly income of 3100.00, (minimum of 300 to a maximum of 12000) (14 of the participants not will define their monthly income).

**Table 1:** Socio-demographic characteristics of four FGDs = 33 and 18 IDIs study participants to Exploring health literacy in relation to non-communicable chronic diseases preventive behavior among adults in Amhara Region, Northwest, Ethiopia, 2025 (N = 51).

S. No.	VariableVariable		Frequency	PercentPercent
1	**Gender**	Male	20	39.2
		Female	31	60.8
		Total	51	100.0
2	**Completed Highest Level of Education**	Cannot read and write	5	9.8
		Below 12th grade	15	29.4
		12th completed	5	9.8
		Certificate plus	26	51.0
		Total	51	100.0
3	**Marital Status**	Single	22	43.1
		Married	22	43.1
		Divorced	4	7.8
		Separated	3	5.9
		Total	51	100.0
4	**Occupation**	Unemployed	13	25.5
		Government Employee	22	43.1
		Non-Government Employee	2	3.9
		Private Employee	3	5.9
		Pension	2	3.9
		Others^*^	8	15.7
		Total	50	98.0
5	**Religion**	Ethiopian Orthodox	50	98.0
		Muslim	1	2.0
		Total	51	100.0

NB. Others*: students, daily laborer, housewives; one participant not defined his/her occupation.

### HL and chronic disease prevention: A multidomain perspective

Participants in this study, represented a diverse demographic in terms of socioeconomic class and educational background. They reported varying levels access to preventive healthcare, barriers to seeking healthcare services, and engagement in common NCD prevention behaviors. By comparing the initial concepts, 135 codes and 21 categories were developed and organized into 9 sub-theme groups. Finally, four themes emerged (details are presented in Table in S2 Table in S1 File). From the IDI and FGD participants the four main themes identified are: 1) health access and barriers, 2), understanding (disease knowledge and awareness), 3) appraisal, and 4) health behavior and decision-making. In this section, quotes from participants are included in the explanations of the sub-themes provided below.

### Theme 1: Health access and barriers

#### Sub-theme 1: Healthcare access.

In this study, participants were asked where they usually found health and medical information. They reported typically obtaining it from health extension workers, the media, or other reading materials from healthcare settings (print media formats). A small percentage of participants, specifically government employees with smartphones, used social media platforms, such as Facebook and the internet (new media), while the rest relied on traditional media, including television. Others depended on community services for information. A participant described this as follows:

*“Normally, it fluctuates depending on the state of society. The majority of our society is unaware/unfamiliar; yet, there are some educated people. Just/properly uneducated individuals – will have an information access from health professionals; other segments of society are more informed and made aware of in various ways via the media or other means. If there is a need to delve deeper (within) and learn, it differs depending on the educated members of society.”* **25 years, M, FGD_1_ P-05**

Another FGD Participant also Stated as:

*“Information can be obtained in a variety of ways. Information can also be obtained through television and radio. When medical experts come to visit us from one home to another, they can potentially spread it to us. We can also obtain it by visiting a medical facility.”* ***28 Years F; FGD_2_ P- 03***

#### Sub-theme 2: Promising opportunities for health literacy.

To enable long-term behavior change, enhance risk comprehension, and close knowledge gaps, participants cited several effective solutions currently in place. These include, weekly health programs on television, social and community interventions, Family Health Teams’ home-based services, volunteer and peer support, free care programs, health insurance, access to fitness centers, plain-language resources, culturally specific campaigns, community-based initiatives such as health education and group exercise), and technological tools like smartphone applications. All were seen as promising ways to increase the adoption of NCD preventive practices and improve information access. The impact of family cues, university-led education, and urban agriculture programs were also mentioned. A participant reported about this as:

*“We all have numerous things that we may do to protect ourselves from these diseases, the majority of us live in areas where too many settlements are there. I reside in a community with houses that are far apart. There is a field, no odor, and nothing else to bother me. Nothing disrupts my peace or causes disease. When you leave your house and travel into the neighborhood, you will find a field, a clean climate, and water that is as clean as possible, which is convenient for you.”* **26 Years, M; P- 7**

While acknowledging the complexity of issue, participants expressed their belief that economic expansion can be an effective strategy for improving overall population health. Similarly, participants suggested increased collaboration between universities and other sectors, emphasizing the need for preventative messages from media and sports teams. They recommended developing programs to enhance community awareness by engaging with community gatherings, religious institutions, and school programs to raise awareness like Human Immune Deficiency Virus/Acquired Immune Deficiency Syndrome (HIV/AIDS). They also mentioned the availability of previously held tea and coffee ceremony at work sites, and proposed strategies for screening (health check-ups) for those who visit healthcare settings but are not currently seeking medical care, as both immediate and long-term solutions for combating NCDs. One participant described this as follows:

*“I believe it is vital to conduct a community-wide awareness-raising initiatives, educate the community about the origins and effects of the diseases condition, and exercise caution. If there is education at home, establish situations that allow family discussions about concerns, and if there is a neighbor, explain the diseases to them, but I believe the most important thing is to raise awareness.”* **38 Years, F; P- 4**

#### Sub-theme 3: Barriers to healthcare.

Access to quality healthcare and reliable health information is critical for maintaining good health, but many systemic and structural barriers prevent people from fully accessing these services, especially among marginalized groups. Barriers related to geography, language, and culture can make it more difficult for individuals to receive preventative services such as health education and screenings, and to navigate the healthcare system and health information for preventive engagement.

**Access related barriers.** In this study, participants identified a verity of barriers that delay their access to common NCD preventive measures, healthcare preventive service, and health information. Even if some screening services are available, they are not well promoted. Almost all study participants noted the lack of a dedicated, comprehensive preventive health services room in any healthcare setting, especially in the primary health care units, which makes it difficult to access specialized preventive services for common NCDs. As one participant stated:

**“***Furthermore, sports facilities, for example, now allow people with specified income levels to participate in sports activities for a fee. Some persons with limited or no income can play sports at home. However, this is insufficient because the lack of adequate and well-organized sports facilities is an issue in and of itself.”* ***30 Years, M; P- 15***

**Sociocultural and language barrier.** Participants reported facing several challenges that make it harder to receive preventive health services, health information, and to engage in preventive measures for disease conditions, thereby affecting their ability to maintain health throughout their lives. Many barriers are individual focused. However, some required legal actions, while others will need to be intervened in. Preventive care is discouraged by healthcare issues such as lengthy wait times, fragmented care, and provider shortages, particularly for people with low health literacy who may find it difficult to understand medical instructions or advocate for themselves. Participants reported a considerable burden of operational failures. Financial difficulties, missed or delayed hospital check-up appointments, lack of physical activity, avoiding walking (using a taxi for short distances), spending too much time watching television, and frequent bar visits were the most common failures related to preventive behaviors for common chronic NCDs outside of their own practice. As one person put it:

*“For us and as individuals, the fundamental issue is a lack of awareness. For example, there is a problem with media mobilization in the media; whether individuals or I want to conduct work (prevention actions), there is nothing comprehensive. There is a severe awareness issue.”* **37 Years, HEW;** P- **11**

**Interpersonal communication (IPC) related barrier.** Without effective communication among healthcare practitioners, the proper delivery of healthcare services may be jeopardized. People with low HL are disproportionately affected by complex healthcare systems and restrictive policies, such as limited insurance coverage and a lack of incentives for preventive care. These individuals often struggle to navigate coverage plans or understand the benefits accessible to them. Almost all FGD and IDI participants shared similar observations; they reported facing various challenges that make communicating with healthcare providers difficult. Common complaints including miscommunication between parties, malingering or truancy, and frequently checking work areas outside of normal hours were also common complaints among participants. One participant in the FGD/ stated:

*“What we discovered was that when we visit a health center, doctors do not devote enough time to each client. This is because, for example, when a client visits a health facility for treatment, it is important to correctly evaluate the person’s entire needs and guarantee that the user receives the care they require”.* **20 Years F; FGD**_**3**_ **P- 02**

Similarly,

*“The professionals forced me to start taking medication right away, but I refused to accept the decision. I said that ‘I can fix it on my own strength, and if not, I should let me do it by coming to this hospital or the facility where I live,’ so I spoke with the health professional and informed him that I would not begin taking medication. The health expert stated that I do not agree with this notion, will not sign it, am unsure, and cannot agree with it.”* **50 Years, Male; FGD**_**4**_ **P-06**

Additionally, social influence and support networks — whether familial, peer, or community-based — can either encourage critical evaluation of health information (such as collective lifestyle changes) or reinforce harmful health behaviors (such as normalization of smoking). Adequate health literacy promotes social inclusion and improves communication, strengthening support systems and interpersonal relationships. In this regard, participants were asked how well they interact with their family, neighbors, healthcare providers, or anyone else knowledgeable to improve their understanding of common NCDs, whether they understood the material they read, possible preventive measures, and common modifiable risk factors. Overall, they limited their interactions about these difficulties, even if they had a healthcare expert in their family. Only people who have been unwell actively seek to improve their habits. Without illness, most individuals have difficulty adopting improved disease prevention behaviors and lifestyles. Almost everyone believes they do not need to know more about these diseases unless they encounter a problem, such as someone contracting the illness. One participant reported this as follows:

***“****We frequently seek remedies after the problem has occurred, as we lack the experience to engage in screening behavior prior to the problem occurring. However, when the disease spreads and infects all sections of society, we are reminded that we must defend ourselves.”* **37 Years, F; P- 5**

**HL-competencies and knowledge related barriers.** Other barriers to obtaining preventive health care services and health information are autonomy and awareness. Knowledge gaps and educational discrepancies exacerbate the problem, making it difficult for people to find reliable health information amid widespread misinformation, compounded by economic problems, weak policies, and a scarcity of trustworthy information sources. These barriers prevent people from gaining the necessary knowledge about chronic illness risks and preventive measures. Participants in this study experienced difficulty accessing health information, which influenced their decision to seek common chronic NCD preventive interventions. Moreover, as reported by some survey participants, a lack of expertise in requesting information about diseases was also a barrier to obtaining health information. One participant in the FGD stated:

*“Starting from myself, I can see the limitations of the activities we do to understand and examine our body and know our own health. Such diseases are very common, but the activities we do to prevent them and inform them are very low; even from a professional standpoint, we see a lot of people from the city to the countryside dying from high blood pressure and diabetes.”* **60 Years, M; FGD**_**1**_ **P – 08**

Some participants hesitated to seek health check-ups due to anticipated fear, embarrassment, and self-stigma. Concerns about others’ perceptions, especially regarding access to common NCD preventive services and health information, compounded this hesitation. Additionally, discrimination, poor attitudes from health workers and delays in service delivery were reported, further discouraging participants from seeking health check-ups. These concerns are illustrated in the following excerpts.

*“We get information. But after reading some, I quit because I am frightened. …, there are always questions over what causes diabetes. What are the methods for preventing it? …. I read the banners and ask myself, “What if it happens to me?” and then I stop reading again. I see people discussing about high blood pressure on social media lately, but I avoid it/don’t read it because I’m terrified. There is a need, but there is fear and something inside you that pushes you; however, I see it directly, and I believe that if I have it, I can have the disease, so I just skip it and don’t read it again till I understand the situation well.”* **30 Years, M;** P-**15**

**Economic and financial related barriers.** Economic and financial considerations play a significant role in shaping HL, particularly in the context of preventing NCDs. Participants in this study, identified a strong relationship between economic growth and health literacy-based health decisions and their consequences. They emphasized that economic development may increase access to critical resources such as a balanced diet and better living conditions, thereby preventing diseases and promoting overall well-being. Participants also identified financial constraints as a barrier to accessing health services and health information, especially since many participants were illiterate and unemployed. Consultation costs, the purchase of healthy food products, and overall cost inflation all contributed to the financial burden for people with variable incomes. Economic and financial considerations introduce survival bias, causing vulnerable populations to deprioritize prevention in favor of competing needs such as housing or food security. These systemic failures exacerbate healthcare challenges, including inconsistent provider messaging and overmedicalized communication, which obscure the risk-benefit analyses essential for prevention. Even when individuals understand health advice, they may be unable to follow it due to cost. Many people sought private health check-ups for NCDs, increasing household expenses. Reflecting on the problems encountered, a participant emphasized:

*“When a person is unable to cope with life due to illness, lack of awareness, or depression, they may lose hope. Now, life is growing more expensive. Everything, and he also suffers from depression. I do not think it is anything else. Second, the person who does not visit a health institution is not covered by health insurance since the cost of health care is prohibitively expensive. He does not have money. The person who also travels privately and visits health insurance providers.”* **60 Years, F;** P- **18**

Another FGD participant said:

*“As a result, we are unable to consume the necessary vegetables and fruits; if we did, we would be able to preserve our health. And the primary issue is the source of money. There are no jobs. The environment is poor. Of sure, ….”* **70 Years, M; P-10**

### Theme 2: Understanding (disease knowledge and awareness)

This research paper examines the complex relationship between knowledge, awareness, and promising opportunities for improving health literacy regarding NCD preventive behavior, specifically how individuals’ understanding and recognition of a problem influence their ability to identify and implement effective solutions. HL (understanding) – is critical for effective chronic disease prevention, as it determines an individual’s ability to process, interpret, and apply health information to make informed decisions. It is shaped by multiple interrelated factors, such as limited formal education, language barriers, misinformation, and complex medical terminology.

#### Sub-theme 1: Health knowledge and awareness.

In this context, only two IDI participants discussed CRD (e.g., COPD, asthma), focusing more specifically on asthma and the four major modifiable risk factors for these illnesses over which they attempted to gain control. A participant described this as:

*“Age is a risk factor; as people get older, they develop issues. Second, ……, because she has not given birth, a woman must give birth. First and foremost, our uterus requires sexual intercourse in order to function properly. I believe that women have difficulties that we can solve by giving birth, breastfeeding, and having sex. Another factor to consider is age; those over 60 are more likely to not bleed/stop menstruating/have high blood pressure or diabetes, and when menstrual flow is interrupted, they are more likely to have a disease.”* **A 30 Years, F; FGD**_**3**_**-P- 07**

Another interviewee participant said:

*A person who is slim does not experience high blood pressure.* ***30 Years, F;*** *P-****1***

Adequate health literacy requires accurate knowledge about chronic diseases and their prevention. Many individuals have gaps in understanding risk factors, symptoms, and preventive measures due to limited education or unreliable information sources. Misinformation or a lack of culturally appropriate materials further widens these gaps. As health extension workers revealed, in the study setting, various activities were implemented to help people adopt healthier lifestyles. Some participants had no idea what a risk factor or preventive behavior for common chronic NCDs is. As one participant descried:

*“…, when it comes to noncommunicable diseases such as diabetes, which are personal diseases, I am no longer in the profession; instead, I am an individual as a member of the community. To be honest, I don’t know how diabetes develops; I only recently learnt. Only my own... What do I have, but in this health state, I’m not sure how diabetes develops. I believe that is simply ignorance.”* **25 Years, M; FGD**_**1**_ **P- 05**

### Theme 3: Appraisal (social and emotional well-being)

Chronic disease risk appraisal depends on accurate, culturally relevant health information. Knowledge gaps arise from limited education, language barriers, or reliance on unreliable sources such as social media. These gaps are also influenced by cultural norms such as distrust of Western medicine or traditional beliefs such as preference for herbal remedies. Behavioral economics further reveals how present bias – the tendency to prioritize immediate rewards over future benefits – undermines engagement in risk reduction measures.

#### Sub-theme 1: Social influence and support.

Social influences, cultural ideas, and strong social support networks affect how people acquire and use health information, which can either help or hinder an individual’s ability to connect with critical health information. Regarding this sub-theme, in this study, many participants discussed the value of having friends or family to provide social support. As some younger participants stated, individuals who have a network of people from whom they can seek guidance and support are more likely to engage in preventive behaviors than older adults who lack such a network (social capital). In contrast, individuals in younger age groups tend to act carelessly and recklessly, do not perceive themselves as vulnerable to disease, and are highly influenced by their peers. As stated by a participant:

*“When we look at our culture, we accept what we are told exactly; we only see it from the standpoint of thinking that professionals are in a better condition; mine came from me, was taught by me, my son, and our society. A person who has been educated to do more, …, to be a creator, ……; that is, to face, not only in health care but in any institution, when you go to the staff, when you see our society, you do not face the service that you use*.” **25 Years, M; FGD**_**1**_ **P- 05.**

#### Sub-theme 2: Stress and emotional health.

Respondents’ answers highlighted the significance of stress levels and emotional states (anxiety, concern, annoyance, worry), which were considered reasons why people do not participate in preventive measures for common NCDs. Nearly all did not engage in preventive practices due to ignorance, bashfulness or sentiment (Yelugneta) that may arise from failing to follow healthcare provider recommendations, and misunderstanding about risks and preventive practices. According to most participants, this mindset was caused by misconceptions and myths. Participants emphasized that stressful life circumstances, including current living conditions, can deter people from taking preventive action. As stated by a participant:

*“The question arises.... Because you don’t comprehend, it adds more stress to your situation. As previously said, if I go to someone’s house and say, “Hey, salty...,” they answer, “No, it doesn’t exist.” When they say this, you accept it because you do not wish to debate. But if you say, “Don’t do this,” because you don’t like me, you’re saying it.”* **21 Years, F;** P- **14**

Participants were also concerned about genetic changes and the use of chemical sprays to increase horticultural and livestock efficiency and productivity. This raises questions about the community’s freedom to use these methods. As described by a participant:

*“All vegetables and fruits on the market have undergone several genetic alterations in order to increase productivity and efficiency. Chemicals are sprayed. This raises problems regarding the community’s freedom to use them. The chemicals they use also have adverse impacts on the customer. The current market is not favorable for our bodies (0:40:42-44); this is encouraging to see. This means that most agricultural experts are now focusing on these veggies in order to enhance vegetable production and productivity, which is creating consumer side effects.”* **24 Years, M; FGD**_**4**_ **P-04**

#### Sub-theme 3: Social and cultural norms.

As described by both FGD and IDI participants, there is a long-standing belief that short-distance driving, using a vehicle, and consuming fatty meats such as mutton are all signs of wealth and good health. In the research locations, participants reported that sedentary lifestyles and the consumption of fatty foods for breakfast, lunch, and dinner were common. We also found high social connectedness and a friendly mindset, which promotes connection, but can also lead to increased behaviors and stress, as well as unpleasant feelings from denying requests to drink and smoke. One participant described this as:

*“……, if you do not drink alcohol with friends before engaging in such scenarios with staff at any institution, you will be regarded as a loner and a money-hungry/stingy/imbecilic individual. These conditions influence you. Furthermore, the proliferation of alcohol-serving establishments has its own set of consequences. Most of your friends’ visits frequently areas where alcohol is sold; you accompany them to spend time with them. There’s peer pressure.”* **30 Years, M; P-15**

Participants discussed the significant influence of traditional, cultural, and religious beliefs, which can encourage some people to change their behavior but can also cause others to accept illness as a natural or spiritual process, reducing the urgency to take preventive measures. Such interactions can jeopardize HL, especially when people struggle to reconcile multiple health paradigms or lack the ability to critically evaluate competing sources of information. For example, participants reported following religious leaders’ advice to visit a baptistery (where they would be bathed in holy water) and to be massed (endiqorbu), and to pray, but they were never advised to stop taking their medication, if any. A participant described this as:

*“If the problem does not arise, we can avoid it by fasting, praying, and communicating with the Creator. This is a form of faith that allows us to be free from difficulties before things has had been occurred.”* **38 Years, F; P- 6**

Additionally, both FGD and IDI participants reported issues related to individuals’ careless attitudes toward health, difficulty in asserting their rights, and inability to adhere to chronic disease risk reduction measures – including initiation, implementation, and discontinuation. Most study participants noted particular difficulty with initiation**.** This stated by a participant:

*“… I haven’t had the experience of taking preventive measures till we are being bed reddened patients, not because I consider myself well, because I want to take care of myself and be healthy. Despite being aware of it, we ignore it. We do not value ourselves.”* ***24 Years, F; FGD***_***1***_ ***P- 01.***

We asked the health extension workers about the participants’ understanding, at the time of the interview, of the community’s knowledge of common NCDs, modifiable risk factors, preventive measures, and behaviors, as well as the reasons the community does not take preventive action and how they seek health information to improve their knowledge. According to the participants, the community’s social and cultural norms significantly influence their overall knowledge of disease conditions and their attitudes toward preventive measures. In the absence of symptoms, no one is interested in learning about disease conditions; even during the family health program, most people are unwilling to check their blood pressure and blood sugar levels. Most are unaware of the benefits of preventive measures and are not motivated to learn about or follow them. A participant described this as:

*“We frequently seek remedies after the problem has occurred, as we lack the experience to engage in health screening behavior prior to the problem occurring. However, when the disease spreads and infects all sections of society, we are reminded that we must defend ourselves.”* **36 Years, F;** P-**5**

Another FGD participant said:

*“People who seek information or assistance regarding diseases that have spread in the community, except while they are sleeping, are also known as pamphlets; they are also known as media; they turn to something else, wondering what it will do for them. They go to hear the newest news, but they do not pay attention to it.”* **30 Years, F; FGD**_**1**_ **P- 03**

#### Sub-theme 4: Perceived risk and vulnerability.

Perceived risk and vulnerability are critical for evaluating preventive measures. Misjudging personal risk (e.g., “I am not susceptible to diabetes”) often results from low HL or misinformation. People, may also underestimate their susceptibility due to misaligned risk perceptions influenced by false information or limited HL, which can delay essential preventive actions.

This study examines how individuals’ awareness of their susceptibility to common NCDs affects their likelihood of engaging in health-promoting behaviors and highlights the role of perception in shaping preventive health practices. Some participants reported understanding the health risks associated with unhealthy behaviors, recognizing potential causes of chronic diseases (such as stress), and appreciating the importance of preventive measures (for example, living in a neighborhood far from the city). As the participant put it,

*“Some alcoholic drinks can seriously harm our health. Using them puts us in danger and causes disorders that are linked to it. We slaughter fatty (full of white meat – ruined) cows throughout the year’s festivals; we utilize this junk, but I don’t use it; I see individuals who use junk a lot becoming ill. I no longer utilize trash to keep myself healthy, though.*” **37 Years, F; Married, Daily Laborer** P- **2**

Similarly, another participant described this as:

*“Now, eating fatty and fried foods increases your risk of contracting these illnesses. A wide range of drugs can also rise blood pressure. If we have a cardiac problem, the final stage of heart disease can also occur. If you repeat it, it is an irreversible problem once it reaches the stage of heart disease. Blood pressure is caused by stress and a lack of proper nutrition; many of us are predisposed to these issues.”* **32 Years, F; FGD**_**3**_**-P- 06.**

Participants also expressed concern about the seriousness of these illnesses, which contributed to their sense of vulnerability and perceived risk. One participant said of the problem:

*“If difficulties arise, the prospects of recovery are minimal. …. They gradually reduce these kinds of addictions. When they are exposed to these addictive chemicals, they become extremely ill, their chances of death increase dramatically, and rehabilitation becomes tough.”* ***35 Years, Daily laborer, F; FGD***_***3***_***-P- 05***

Poor risk assessment, driven by a lack of awareness or a low sense of vulnerability, may delay timely action and worsen outcomes. However, many participants stated they were unaware of the importance of preventive health and did not fully understand risk factors, leading to misunderstanding about risks and a diminished sense of vulnerability. Regarding the matter, one participant said:

*“I frequently walk like a bee. I attribute this to God’s gift of grace. I work till I’m tired and sweaty. If you ask my sister, she will not move. If you ask me what occurred, she will not be able to work. What is the issue with her? She is gaining weight and is unable to work; what is the problem with her lack of exercise. We used to walk from this neighborhood to the general secondary school (about 7-9 kilometers away) and play intense sports without realizing it. As previously stated, the taxi arrived; nonetheless, this is hazardous to one’s health.”* ***41 Years, Divorced; F, FGD***_***2***_ ***P- 06***

We were asked about the prevalence of sickness in relation to gender differences, and the participants stated that women are a more vulnerable demographic. One participant commented on the issue that:

*“Being a woman makes her susceptible to various ailments. Why? How? Because a woman is frequently under pressure; because she is under pressure from both housework and outside work, a man is frequently a waste who does not notice it (does not think much about anything) and goes to sleep, whereas a woman takes care of everything; first there is childbirth, then there is breastfeeding, and then there is a lot of housework at home; in all of this, the woman makes more sacrifices than the man. As a result, the lady is exposed first.”* ***38 Years, Housewife; F, FGD***_***3***_ ***-P- 07***

### Theme 4: Health behavior and decision-making

#### Sub-theme 1: Healthy behavior.

Our empirical data indicate a relationship between behavioral factors and health literacy regarding preventive measures for common NCDs. Some participants reported exercising at home as much as their standard of living allowed. They also tried to improve their meals and family time, included fruits and vegetables in their diet, used salt sparingly, participated in extracurricular work activities, attended community events, and used media and technology for health management and health information. Other individuals visited healthcare setting for routine check-ups (mostly females for cervical cancer screening), while some routinely engaged in activities such as cleaning the byre or cattle pen at home, avoiding sweets, and not going to drinking establishments. One participant stated that:

*“……, alcoholic beverages, particularly the Habesha Areki drink (local alcohol), are extremely dangerous. Third, imported packaged beverages are... The so-called soft drinks, whose nature is unknown, are used to ask a patient when he is unwell; and during birthing, we do not know their nature; they are pleasant, but if we have such issues, I dislike, I do not drink them; instead, I drink tea and occasionally Tela (local/homemade alcohol)-What is made in a conventional manner (locally prepared bear drinks like – Tela), I drink it in moderation and do not consume anything else. And I believe it is best not to utilize such imported items. We do not understand their nature. They are ready for trade, which is not good. I believe they can also cause sickness. Also, drinking and smoking are bad. I believe so.”* A **70 Years, M;** P- **10.**

Another FGD Participant added as:

*“I frequently walk like a bee. I attribute this to God’s gift of grace. I work till I’m tired and sweaty. If you ask my sister, she will not move. If you ask me what occurred, she will not be able to work. ….. She is gaining weight and is unable to work; …. We used to walk from this neighborhood to the general secondary school (about 7-9 kilometers away) and play intense sports without realizing it. As previously stated, the taxi arrived; nonetheless, this is hazardous to one’s health.” A* **41 Years, F; FGD_2_ P- 06**

Most people had not led healthy lives before experiencing health issues. To protect themselves from disease, they self-medicate. Young people mostly went to bars, especially on weekends, and participated in sports only intermittently. They added that they prioritized daily duties and necessities over following healthcare providers’ advice on preventive measures. As highlighted by one FGD participant:

*“……. It signifies it contains a lot of things. But what I’d want to point out is that there is a person who is unwell but does not go to the medical center or get checked because of work overload, an unwillingness to listen to himself, or death. Recently, I had a tailor as a neighbor. Recently, a neighbor of mine became a tailor; he departs in the morning and returns home at night; when he first started, he claimed to have a cold, but he just utilized hot liquids prepared at home and departed in the morning. He had no rest and eventually went to the hospital under our pressure, where he was diagnosed with a lung condition.”* **37 Years, M; FGD**_**4**_ **P-07**

#### Sub-theme 2: Decision-making and adherence dynamics.

In this sub-theme, the association between decision-making processes, adherence to health advice, and the adoption of preventive practices for common NCDs was explored. According to both FGD and IDI participants, factors that influencing people’s decisions about common chronic NCDs prevention activities include healthcare provider recommendations, awareness levels, willingness to visit a healthcare facility for a check-up, perceptions of the severity of chronic illnesses, and health knowledge and attitudes. A lack of knowledge, financial concerns, social networks, neglect of self-care, and insufficient skills to evaluate or make informed decisions about their health are all cited as contributing factors to poor choices. As highlighted by a participant:

*“First, we lack knowledge; society is unaware, and there is no situation in which it guesses there are difficulties that could affect me. Because it is expected that everything is tranquil, this type of mindset must be corrected/eradicated.”* **70 Years, Retired M;** P- **10.**

### Participants’ level of health literacy

By highlighting the contextual, cultural, and interpretive aspects of health-related behaviors, can lead to a better understanding of HL in relation to chronic disease prevention behavior. This study examines how people interpret, negotiate, and implement preventive behaviors – such as healthy eating, following preventive measures, exercising, and getting regular check-ups – that are influenced by systemic barriers, sociocultural norms, and lived experiences rather than just knowledge acquisition. Rather than a simple lack of knowledge, a person’s unwillingness to adopt NCD prevention measures may stem from culturally ingrained beliefs about illness, financial constraints, or mistrust of medical institutions. Similarly, gendered norms that discourage prioritizing self-care, along with the collective memory of medical exploitation, can influence preventive behaviors for NCDs. Developing treatments for chronic disease prevention requires approaches that are not only understandable but also culturally appropriate, and not only educational but also empowering within the social norms of the communities they serve.

Health literacy can evolve from a patient education tool to a lever for equality and lasting behavior change. By comparing how patients, families, and clinicians assign different meanings to illness – revealing mismatches that undermine prevention – this perspective offers a transformative lens for understanding preventive behavior for chronic diseases. It also highlights how preventive behaviors are negotiated within dynamic contexts, where factors such as structural racism, economic precarity, cultural stigma, and historical trauma shape decision-making in ways that go beyond individual “compliance,” exposing the invisible social scripts and power dynamics that standard interventions often overlook.

## Discussion

Participants in the study came from diverse educational and socioeconomic backgrounds, and the results showed notable differences in healthcare access as well as challenges in implementing preventive strategies for common NCDs. Major obstacles included communication issues, lack of awareness, limited access to information, and the absence of private rooms for health check-ups. A few participants who have a contact with someone who experienced disease conditions were well-informed about the causes of chronic disorders and available preventive services, while others, particularly those at the extremes of age, showed a lack of understanding regarding risk factors and preventive strategies. The study also examined the relationship between adopting preventive health practices and social and emotional well-being. Four primary themes emerged: health access and barriers, disease knowledge and awareness, appraisal, and health behavior and decision-making.

Health literacy was essential for individuals to access healthcare and overcome obstacles. Those with higher HL were better able to seek out health information and preventive services, which they believed could help prevent NCDs and promote healthier lives. However, many participants – especially those from disadvantaged groups – struggled to find trustworthy health information and services. These findings are consistent with a study conducted in Eastern Ethiopia, which identified similar obstacles to heart disease awareness and prevention practices [30]. Barriers to preventive healthcare highlighted by participants including lack of physical activity, frequent bar visits, and limited knowledge about screening services and risk factors for NCDs. These difficulties were exacerbated by time constraints and cultural beliefs. Many participants stated that healthcare facilities were frequently overworked and that preventive health interventions were either unavailable or insufficiently provided. Bulto et al.‘s 2022 research [30], which observed similar challenges in Ethiopian healthcare settings, supports this finding. This may be because medical facilities are overburdened with patients and have limited time to devote each individual. However, health care structures might not be a problem in the Ethiopian context

Participants’ knowledge and opinions regarding NCDs and associated risk factors varied widely. Some, such as those with internet access or frequent contact with HEWs, were well-informed, while others such as older or unemployed individuals exhibited prejudices and misunderstandings, especially regarding avoidable risk factors. This finding contradicts studies done by Hareru HE, et al. (2024) [31] and Bulto LN, et al. (2022) [30] which found that patients were reasonably aware of risk factors for heart disease and understood the risks of smoking shisha. The degree of interaction with healthcare professionals during appointments and differences in study settings could explain this disparity. Additionally, people who encounter problems often seek more information about topics of interest; these challenges may help individuals gain a better understanding. Despite the HEWs’ efforts, a few participants mentioned that they occasionally attend health education sessions. This result is consistent with a qualitative study conducted by Pilusa T.D et al. (2025) [32]. However, unlike the studies conducted in Iran [33] and Ethiopia [34], participants in this study limited their conversations to family members, communities, medical professionals, and other knowledgeable individuals – even if they had a healthcare provider in their home. Despite these obstacles, healthcare professionals encouraged people to adopt healthier habits by actively participating in health education initiatives. Instead of seeking official health education, participants frequently turned to their family, friends, and medical professionals for information. This research emphasizes the need for policymakers and health services to abandon a “one-size-fits-all” approach and create customized solutions for people who are socioeconomically disadvantaged.

Aligning with the framework for health-literate systems proposed by Sørensen et al. [35], the study also underlined the necessity of health literacy-responsive services. These services should be jointly developed to take into account various cultural settings, social norms, and health literacy requirements. Responsive health systems can decrease exposure to NCD risk factors caused by misinformation, increase access to health information, and advance health equity [20]. According to participants, the adoption of preventive health behaviors is significantly influenced by cultural norms, social support systems, and emotional well-being, all of which greatly affects their willingness to take precautions and reduce risk. HEWs also reported that participants’ social and cultural norms have a significant impact on their attitudes towards preventive action and their understanding of disease conditions.

Additionally, there are widespread traditional beliefs about the benefits of consuming high-fat, using vehicles for short distances, and drinking factory-made beverages. These findings are in line with a study by Bulto LN et.al. [30] which found that consuming meat, particularly white meat, is considered a sign of affluence and high status. Similarly, a study conducted in public hospitals in the region revealed that cultural traditions within families and social ceremonies compel individuals to consume prohibited foods and beverages that worsen their health conditions, thereby influencing adherence to preventive health behaviors [36]. Studies conducted in Kenya [37] have also confirmed this, indicating that low HL levels result from cultural norms and misinformation. According to the study, to enhance preventive behaviors across all age groups and cultural contexts, future HL programs should consider social and emotional well-being and establish support networks.

The study also found that the main determinants of individuals decisions to adopt preventive health practices were perceptions of the severity of the condition, awareness levels, and recommendations from healthcare providers. Participants who felt more susceptible to NCDs were more likely to adopt health lifestyle choices. A recent scoping review by Ho et al. [38] supports this finding. This result is also consistent with studies conducted in Ethiopia [31], which found a strong correlation between individuals willingness to take preventive action and their perceptions of risk. These findings suggests that factors influencing health behaviors – such as healthcare provider recommendations, awareness, perceptions of risk, and social support -highlight the importance of empowering individuals with the knowledge and tools needed to make informed decisions about disease prevention and healthy lifestyle practices within the context of HL.

### Implication

Research on HL, NCDs, and preventive behavior has important implications for future research, practice, and policy. By addressing the burden of NCDs, supporting preventive behaviors, and increasing HL, stakeholders can improve individual and population health outcomes. Enhancing health literacy at the community level enables people to take preventive measures and make informed decisions about their health. By providing accessible, culturally appropriate health information and resources, community health professionals and neighborhood organizations can make a significant contribution.

HL is one of the three pillars in the WHO Shanghai Declaration on Health Promotion (2016) for achieving sustainable health development by 2030 [39]. It is also included in WHO NCD-related policy documents to guide policies, programs, and interventions for NCDs to be more effective and reach more people [40]. From a policy perspective, investigating community HL provides insight into who is being left behind and who is not accessing or benefiting from health services. Community-level efforts can also combat NCDs by encouraging preventive behaviors and fostering conditions that promote healthy living. Prioritizing these programs allows communities to reduce risk factors for NCDs, such as physical inactivity and poor nutrition, while fostering a culture of health and prevention.

In the future, community-based studies can examine how HL and preventive behaviors affect NCD outcomes in specific populations. Researchers can collaborate with local organizations to develop and test interventions that address the community’s specific needs, such as peer-led education programs or mobile health clinics. By focusing on community-level solutions, research can offer valuable insights for reducing health disparities, promoting equitable access to care, and ultimately lowering the burden of chronic diseases. Researchers should continue seeking innovative solutions to these complex challenges, and policymakers should prioritize equitable and evidence-based approaches. A multidisciplinary strategy that integrates prevention and HL into NCD management can lead to healthier, more resilient communities.

### Strength and limitation

This is the first qualitative community-level study to explore HL regarding NCD preventive behaviors, as well as NCD-specific knowledge and beliefs of among adults in the Amhara Region, Northwest Ethiopia. The explicit strength of this study is its focus on the community level and health extension workers – who are integrated within communities and work directly with groups that are often disadvantaged or marginalized. This approach provides comprehensive insights from a grassroots perspective into HL challenges and practices. This bottom-up method enables a thorough understanding of lived experiences, cultural nuances, and regional barriers to HL that may be overlooked by higher-level practitioners. It helps to amplify the voices of those most directly involved in everyday health promotion and education. The study findings add depth and meaning to the existing knowledge. This study also enhances our understanding of the diverse HL challenges that adults in the Region face when trying to access, understand, and use health information and preventive health services to actively manage their health and maintain healthy lifestyles. Furthermore, this study provides a knowledge template that may be applicable to other contexts nationally and internationally (especially in low- and middle-income countries) to better understand HL and the broader needs of the adult population at the community level.

The main limitation of this study is the use of a purposive sampling strategy to recruit adults from a subset of the entire community. This resulted in an overrepresentation of adults from urban areas with greater access to information and healthcare, introducing selection bias. It is likely that adults in rural community, who have lower educational attainment, limited access to health information, and higher levels of social connectedness influencing their beliefs, as well as socio-economic disadvantages, may face more significant HL challenges than those included in this study. Furthermore, interviews did not include healthcare providers other than HEWs and did not encompass the perspectives of professionals and health system leaders, particularly those involved NCD prevention and SBCC coordination offices. This omission results in missing systemic and policy-level perspectives, such as program design and support systems for HL initiatives, training and resource allocation for HEWs, and barriers at the structural or administrative level that impact health communication efforts on the ground. These gaps affect the ability of HEWs and community members to improve HL.

## Conclusions

In summary, this study reframes HL as a social practice rather than a personal competency. To prevent chronic diseases, interventions must: (1) decode the symbolic meanings of behaviors; (2) remove systemic barriers that distort health communication; and (3) democratize knowledge creation by emphasizing community epistemologies. The findings reinforce that most lifestyle factors are within individuals’ control and thus, urge the health system and policymakers to focus on addressing the broader social, ecological, and cultural determinants of health to support individuals in the study setting in adopting healthy lifestyle practices for themselves and their families. The study highlights the need to prioritize HL development at both the individual and community levels to address the evolving needs of individuals and beyond. Such a focus will support, empower, and enable participants and the entire community to reduce the impact of NCDs in the study setting. Specifically, the research reinforces that various risk factors are interactive and can be collectively addressed by focusing on HL. Therefore, future research should engage a more diverse sample of adults or focus specifically on disadvantaged populations using multiple recruitment methods tailored to participants’ needs. This approach will help determine whether their NCD preventive behaviors and knowledge regarding modifiable common risk factors and HL needs are comparable, and will more effectively gather their perspectives to co-design relevant and responsive solutions for these understudied adults.

## Supporting information

S1 File S1 Table: COREQ Checklist. Consolidated criteria for reporting qualitative research (COREQ) checklist for the study on health literacy regarding non-communicable diseases among adults in the Amhara Region, Northwest Ethiopia. S2 Table: Qualitative Analysis Framework. Summary of the emerged themes, sub-themes, categories, and codes, including detailed descriptions of themes and sub-themes related to health literacy and non-communicable disease preventive behaviors.
(ZIP)
