# Supplementary material for: Health literacy on non-communicable diseases prevention among adults in Northwest of Amhara region, Ethiopia: A qualitative study
Source: PLoS One. 2026 Aug 13;21(8):e0354160. doi: 10.1371/journal.pone.0354160 (PMC13472400; doi:10.1371/journal.pone.0354160)
Supplement: Supplementary file 1 [file pone.0354160.s002.docx]

**Point-by-Point Edits Response** on our submission for ***format inaccessibility*** and ***recruitment period writing format in DD/MM/YYYY*** raised by the editorial managers’

**The 1^st^ issue raised by the Editors was about** inaccessibility of original ethics approval document format

**Yes;** the files are uploaded as file type **‘others’:** all the approval documents received from ethics committee of Jimma University to cover the entire range of the study period (i.e. scanned original approval document) and a support letter obtained from East Gojjam Zonal Health Department (Amharic version and the translated - an English Translation) uploaded (stated in the method section of Ethical consideration ___ **Page 8 of 33; Line numbers 213 -214)**. The **‘others** and **supplemental’** files are RAR zipped files and be opened with WinRAR file explorer.

The 2^nd^ one that was raised by the editors also to state the recruitment period for this study to be reported in the Methods section in DD/MM/YYYY format.

Thank you again for the issue you raised; this study is a qualitative in-depth interview and focus Group Discussions approach to collect a data from participants - In this regard we stated the recruitment period for this study as the intended format and ___ **(stated in the method section of Study procedures ___ Page 5 of 33 : line 134- 135)**.
