# Supplementary material for: Health literacy on non-communicable diseases prevention among adults in Northwest of Amhara region, Ethiopia: A qualitative study
Source: PLoS One. 2026 Aug 13;21(8):e0354160. doi: 10.1371/journal.pone.0354160 (PMC13472400; doi:10.1371/journal.pone.0354160)
Supplement: Supplementary file 2 [file pone.0354160.s001.zip › S1_file.pdf.pdf]

S1 Table: COREQ Checklist. Consolidated criteria for reporting qualitative research (COREQ) checklist for the study on health literacy regarding non-communicable diseases among adults in the Amhara Region, Northwest Ethiopia.

COREQ (COnsolidated criteria for REporting Qualitative research) Checklist

A checklist of items that should be included in reports of qualitative research. You must report the page number in your manuscript where you consider each of the items listed in this checklist. If you have not included this information, either revise your manuscript accordingly before submitting or note N/A.

Topic	Item No.	Guide Questions/Description	Reported on Page No.
**Domain 1: Research team and reflexivity**			
Personal characteristics			
Interviewer/facilitator	1	Which author/s conducted the interview or focus group?	Principal Investigator (PI)
Credentials	2	What were the researcher's credentials? E.g. PhD, MD	1MPH, 3 PhD
Occupation	3	What was their occupation at the time of the study?	Accademia, Researchers, *Research Scientist*
Gender	4	Was the researcher male or female?	Both
Experience and training	5	What experience or training did the researcher have?	*Associate Professor, Research Scientist*
Relationship with participants			
Relationship established	6	Was a relationship established prior to study commencement?	P6; the town health extension workers were served as a formal community gatekeeper. The researchers contacted the health centres and health extension works for participants' recruitment and consented for participation on a voluntary basis
Participant knowledge of the interviewer	7	What did the participants know about the researcher? e.g. personal goals, reasons for doing the research	P8; A short discussion took place before each FGDs & IDIs to explain the process and provide clarification.
Interviewer characteristics	8	What characteristics were reported about the inter viewer/facilitator? e.g. Bias, assumptions, reasons and interests in the research topic	PI; MPH
**Domain 2: Study design**			
Theoretical framework			
Methodological orientation and Theory	9	What methodological orientation was stated to underpin the study? e.g. grounded theory, discourse analysis, ethnography, phenomenology, content analysis	P6, Descriptive qualitative study, thematic analysis
Participant selection			

Sampling	10	How were participants selected? e.g. purposive, convenience, consecutive, snowball	P5; criterion based purposive sampling was used
Method of approach	11	How were participants approached? e.g. face-to-face, telephone, mail, email	P6; face-to-face
Sample size	12	How many participants were in the study?	P5, 8: four FGDs comprising 7-10 participants & 18 IDIs
Non-participation	13	How many people refused to participate or dropped out? Reasons?	NA
Setting			
Setting of data collection	14	Where was the data collected? e.g. home, clinic, workplace	P6: in a location that was convenient for the participants
Presence of nonparticipants	15	Was anyone else present besides the participants and researchers?	P5: Two professionals participated during the FGDs
Description of sample	16	What are the important characteristics of the sample? e.g. demographic data, date	P4, 5, & 8: community dwellers, individuals who are working a Kebele level who supporting the health extension program, and health extension workers
Data collection			
Interview guide	17	Were questions, prompts, guides provided by the authors? Was it pilot tested?	P5: Based on the conceptual model of the HLS-EU, a semi-structured FGD and IDI guides, the research team (NM, YK, EG and SB) was developed
Repeat interviews	18	Were repeat inter views carried out? If yes, how many?	NA
Audio/visual recording	19	Did the research use audio or visual recording to collect the data?	P7: Each IDIs and FGDs were recorded with a digital audio recorder. As much as feasible, recordings were made in tranquil environments
Field notes	20	Were field notes made during and/or after the interview or focus group?	P7: notes were collected accordingly
Duration	21	What was the duration of the inter views or focus group?	P6 : Each FGD lasted approximately 1hr and the IDIs lasted with a minimum of 30 minutes
Data saturation	22	Was data saturation discussed?	NA
Transcripts returned	23	Were transcripts returned to participants for comment and/or correction?	P7: the transcribed data, interpretations, and conclusions were returned to a few participants in order to correct errors and challenge what were thought to be incorrect interpretations (member checking)
**Domain 3: analysis and findings**			
Data analysis			

Number of data coders	24	How many data coders coded the data?	P6: independent coder (NM) and validated by (YK)
Description of the coding tree	25	Did authors provide a description of the coding tree?	P6: codes were inductively clustered into initial concepts, categories, Sub-themes, and themes that were compared and discussed to yield an overview of the trends in the views regarding health literacy in the study setting put forward by the study participants.<br>P9: Overall, by comparing the initial concepts, 135 codes and 21 categories were developed and sub-thematized by 9 sub-theme groups
Derivation of themes	26	Were themes identified in advance or derived from the data?	P7: Themes were developed iteratively and reviewed in relation to the research questions<br>P9: Overall, by comparing the initial concepts, 135 codes and 21 categories were developed and sub-thematized by 9 sub-theme groups
Software	27	What software, if applicable, was used to manage the data?	P7: Atlas Ti 9.1.3.0 was used to organize and manage data
Participant checking	28	Did participants provide feedback on the findings?	P7: the transcribed data, interpretations, and conclusions were returned to a few participants in order to correct errors and challenge what were thought to be incorrect interpretations (member checking)
Reporting			
Quotations presented	29	Were participant quotations presented to illustrate the themes/findings?<br>Was each quotation identified? e.g. participant number	P9-25: Result section
Data and findings consistent	30	Was there consistency between the data presented and the findings?	P9-28
Clarity of major themes	31	Were major themes clearly presented in the findings?	P9, 16, 18, & 23: health access and barriers, 2), understanding (disease knowledge and awareness), 3) appraisal, and 4) Health behavior and decision-making
Clarity of minor themes	32	Is there a description of diverse cases or discussion of minor themes?	P9-33

Developed from: Tong A, Sainsbury P, Craig J. Consolidated criteria for reporting qualitative research (COREQ): a 32-item checklist for interviews and focus groups. *International Journal for Quality in Health Care*. 2007. Volume 19, Number 6: pp. 349 – 357

Once you have completed this checklist, please save a copy and upload it as part of your submission. DO NOT include this checklist as part of the main manuscript document. It must be uploaded as a separate file.
