# Supplementary material for: Health literacy on non-communicable diseases prevention among adults in Northwest of Amhara region, Ethiopia: A qualitative study
Source: PLoS One. 2026 Aug 13;21(8):e0354160. doi: 10.1371/journal.pone.0354160 (PMC13472400; doi:10.1371/journal.pone.0354160)
Supplement: Supplementary file 2 [file pone.0354160.s001.zip › S2_file.pdf.pdf]

S2 Table: Qualitative Analysis Framework. Summary of the emerged themes, sub-themes, categories, and codes, including detailed descriptions of themes and sub-themes related to health literacy and non-communicable disease preventive behaviors.

S. No.	Themes	Theme Description	Sub-themes	Description	Initial concepts
	**Theme 1: Health Access and Barriers**	This theme includes the factors that affect access to healthcare, including financial constraints and systemic barriers to care	Healthcare Access	is the ability of a person to identify, comprehend, evaluate, and use healthcare services to suit their health needs. Access isn't only about being able to go to a place or afford it; it's also about being able to grasp information, talk to healthcare providers clearly, and get around the healthcare system.	
			Promising Opportunities for health literacy	examines the economic factors, such as income, insurance coverage, and healthcare costs, that impact health behaviors, access, and decisions.	Promising Opportunities and Solutions
			Barriers to Healthcare<br>• HL-competencies and knowledge related barriers<br>• Sociocultural and language barrier<br>• Access related barriers<br>• Interpersonal communication (IPC) related barrier	This theme focuses on the obstacles or challenges individuals face in accessing healthcare, such as financial, logistical, or structural barriers.	Barriers to Healthcare,<br>Healthcare Challenges,<br>Healthcare System and Policies<br>Economic and Financial Considerations

	Theme 2: Knowledge and Awareness (Understanding)	Description: This theme addresses the understanding individuals have about their health and healthcare options, and the potential solutions to improve health outcomes.	Health Knowledge and Awareness	This group focuses on understanding and accessing health-related information, as well as gaps in education and knowledge that may hinder informed decision-making.<br>.	Health Knowledge and Awareness,<br>Knowledge Gaps and Educational Barriers,<br>Information Sources<br>Knowledge and Information Gaps,
	Theme 3: Appraisal (Social and Emotional Well-being)	Description: This theme focuses on the impact of social relationships, support, and emotional health on overall well-being and health decisions.	Social Influence and Support	This theme explores the influence of cultural and societal expectations, traditions, and values on health beliefs, behaviors, and practices.	Social Influence and Support,<br>Motivational Factors
			Stress and Emotional Health	Emphasizes the mental and emotional aspects of health, including the effects of stress, anxiety, and overall emotional well-being on health outcomes.	Stress and Emotional Health
			Social and Cultural Norms	This theme explores the influence of cultural and societal expectations, traditions, and values on health beliefs, behaviors, and practices.	Cultural and Social Norms,<br>Traditional Health Beliefs and Practices
			Perceived Risk and Vulnerability	This theme addresses the extent to which individuals perceive themselves as at risk for health issues or vulnerabilities, which can influence their health behaviors.	Perceived Risk and Vulnerability,<br>Health Risk Perception,<br>Risk Reduction Measures
	Theme 4: Health and health Decision	Focuses on behaviors and decisions related to health, including	Behavioral Factors	This theme involves the actions, habits, and decisions individuals make that directly impact their health,	Behavioral Factors,<br>Negative Health Behaviors

		health-seeking behaviors, lifestyle choices, and adherence to medical advice.		including lifestyle choices, health behaviors, and self-care practices.	
			Decision-making and Adherence	This theme deals with the processes individuals go through in making health-related decisions, including how they follow through with treatment recommendations and health advice.	Decision-making,
					Healthcare Recommendations and Adherence
