# Supplementary material for: Health literacy on non-communicable diseases prevention among adults in Northwest of Amhara region, Ethiopia: A qualitative study
Source: PLoS One. 2026 Aug 13;21(8):e0354160. doi: 10.1371/journal.pone.0354160 (PMC13472400; doi:10.1371/journal.pone.0354160)
Supplement: Supplementary file 3 [file pone.0354160.s003.docx]

**Response to Editor and Reviewers**

**Manuscript ID:** PONE-D-25-39115

**Title:** Health literacy on non-communicable diseases prevention among adults in Northwest of Amhara region, Ethiopia: a qualitative study

**Journal:** PLOS ONE

Dear Academic Editor Dr. Buna Bhandari,

We thank you and the reviewers for the thoughtful and constructive comments provided on our manuscript. We have carefully revised the manuscript in accordance with all recommendations. Below, we provide a detailed, point-by-point response addressing each issue raised by the editor and reviewers. All changes are reflected in the revised manuscript with track changes. A clean version is also provided.

**1. PLOS ONE *Style and Formatting* requirements**

**Response to Editorial Requirements:** *We have revised the manuscript to meet PLOS ONE formatting requirements, including section organization, citation style, file naming, and figure/table formatting. All files now follow the journal's naming conventions.*

2. In the ethics statement in the Methods, you have specified that verbal consent was obtained. Please provide additional details regarding how this consent was documented and witnessed, and state whether this was approved by the IRB.

**2. Ethics Statement: Verbal Consent Documentation**

**Response:** We expanded the Methods section to clarify how verbal consent was documented and witnessed. Specifically, Verbal consent was obtained and audio-recorded at the start of each interview. Participants verbally acknowledged that they understood the study purpose, procedures, risks, and confidentiality protections. The audio-recorded consent was supplemented by a consent log completed by the data collector and countersigned by an independent witness. The Institutional Review Board approved the procedure for documenting verbal consent.

3. In the online submission form you indicate that your data is not available for proprietary reasons and have provided a contact point for accessing this data. Please note that your current contact point is a co-author on this manuscript. According to our Data Policy, the contact point must not be an author on the manuscript and must be an institutional contact, ideally not an individual. Please revise your data statement to a non-author institutional point of contact, such as a data access or ethics committee, and send this to us via return email. Please also include contact information for the third-party organization, and please include the full citation of where the data can be found.

**3. Data availability contact point**

**Response:** We revised the Data Availability Statement to comply with PLOS ONE’s policy. We replaced the author contact with a non-author institutional data access committee. Full institutional contact details and citation of where the data are stored have been added. “Due to the sensitive nature of the qualitative interview data and the risk of deductive disclosure of participant identity, the full transcripts cannot be shared publicly. Data access is therefore restricted. Researchers who meet the criteria for accessing confidential data may request access through the Institutional Data Access Committee of Debre Markos University, which is independent of the authors. Data can be requested by contacting: **Debre Markos University Data Access Committee**
Email: [**HRCS@dmu.edu.et**](mailto:HRCS@dmu.edu.et) P.O. Box 269 or Office Number: +251588914913.

The committee will review data requests in accordance with institutional and ethical guidelines. The dataset is stored in the secure institutional repository at Debre Markos University

4. Please include captions for your Supporting Information files at the end of your manuscript, and update any in-text citations to match accordingly. Please see our Supporting Information guidelines for more information: [http://journals.plos.org/plosone/s/supporting-information](http://track.editorialmanager.com/CL0/http:%2F%2Fjournals.plos.org%2Fplosone%2Fs%2Fsupporting-information/1/010f019b0ce53c41-8807fdee-3cf6-4a3a-8ee0-638bf685264e-000000/xe_aFbVl7pmcmoAPDw8XCVGTb5XWvAV6PhllH9NdgbM=241).

**4. Captions for Supporting Information**

**Response:** We added full captions for all Supporting Information materials at the end of the manuscript and ensured all in-text citations align with updated file names.

5. We are unable to open your Supporting Information file [Supplemental Files Ziped.rar]. Please kindly revise as necessary and re-upload.

**5. unreadable supporting Information file**

**Response:** We corrected the previously unreadable file (Supplemental Files Ziped.rar) and re-uploaded the Supporting Information in accessible formats (PDF/Word as applicable).

6. If the reviewer comments include a recommendation to cite specific previously published works, please review and evaluate these publications to determine whether they are relevant and should be cited. There is no requirement to cite these works unless the editor has indicated otherwise.

**6. Reviewer-suggested citations**

**Response:** We evaluated all suggested publications and included citations where relevant to enhance the manuscript’s contextual background.

**Reviewer's Responses to Questions/Comments to the Author**

**Reviewer #1:** The authors conducted a qualitative study to investigate health literacy and its influence on NCDs preventive behaviors, providing insights into barriers and facilitators for NCD prevention in the Ethiopian context. The topic is important and may contribute to the development of future health promotion policies in Ethiopia. However, I have some concerns about the methods. Additionally, the manuscript needs significant editing for language and writing quality.

**B. Response to Reviewer 1 (Major Issues)**

**Major Comments**

1. Line **112-118:** Please clarify why these two town administrator sites were selected? Are these areas representative of similar towns in Ethiopia?

**Comment 1: Justification of site selection - clarify why the two town administration sites were selected and whether they are representative.**

**Response:** Thank you for this comment. We have revised Lines **101–111** to clarify the rationale for site selection. We have now added a detailed explanation in the Methods section. The two town administration sites were purposively selected because they are typical medium-sized urban centers in the East Gojjam Zone. These towns have comparable public health facilities, population structures, to capture variation in population characteristics, including differences in education level, economic status, access to health information sources, logistical feasibility, and established primary healthcare units offering NCD screening and health education services. This diversity allowed for a richer understanding of how health literacy influences NCD preventive behavior across different social groups and they maintain accessible health facilities that routinely interact with adult populations. These characteristics made the sites appropriate for examining how health literacy influences preventive behaviors among adults.

2. Line 129-134: How was the sample size determined? And the inclusion/exclusion criteria are not well explained.

**Comment 2: Sample size determination and inclusion/exclusion criteria unclear.**

**Response:** We thank the reviewer for this important comment. We have revised Lines **126–138** to clarify both the rationale for sample size determination and the inclusion and exclusion criteria. In line with qualitative research principles, the sample size was guided by data saturation rather than a predetermined numerical target. Participants were recruited until no new themes emerged from the interviews. We have also clearly specified the inclusion and exclusion criteria to improve transparency and methodological rigor.

3. Methods: Please explain what measures were taken to ensure that the participants adequately represented different demographic groups.

**Comment 3: Representation of demographic groups - Measures taken to ensure demographic representation.**

**Response:** We thank the reviewer for this thoughtful comment. We have revised the Methods section to clarify that, consistent with qualitative research principles, the study did not aim for statistical representativeness but rather for diversity of perspectives relevant to the research question. To achieve this, we used purposive sampling to ensure variation across key demographic and contextual characteristics, including [e.g., age, gender, professional role, years of experience, and administrative setting]. This approach was intended to capture a broad range of experiences and viewpoints, thereby strengthening the depth and credibility of the findings. We have clarified this rationale in the revised manuscript.

4. Results: The results section is somewhat extensive. It would be helpful if the authors could highlight the main themes and findings in a summary table. “

**Comment 4: Long results section- results section lengthy; request for a summary table.**

**Response:** We thank the reviewer for this helpful suggestion. We have condensed the narrative in the Results section to improve clarity and reduce redundancy. In addition, we have included a summary table that highlights the major themes, corresponding sub-themes, and illustrative participant quotations. This table is now provided as Supplementary Table **[S2_file.pdf]** in an accessible format. We note that an earlier version of the supplementary file may not have been accessible due to compression; this has been corrected in the revised submission.

5. I strongly recommend careful English language editing to improve grammar, syntax, and overall clarity. There are several grammatical errors and long, complex sentences throughout the manuscript, which reduce the overall clarity and readability.

**Comment 5: English language and writing quality.**

**Response:** The manuscript has undergone thorough English language revision to improve grammar, clarity, and readability. Long and complex sentences were simplified.

**C. Response to Reviewer 1 - (Minor Issues)**

1. Abstract: “aims to fill” (not “fills”).

**Comment 1: Abstract should use "aims to fill" instead of "fills".**

**Response:** Corrected.

2. **Comment 2: Grammatical error in the abstract -** Grammatical mistake in the sentence “The study used … was conducted …”

**Response:** The sentence structure was corrected for clarity.

3. Line 225: What does “30 years (Range = +/- 50)” mean? Does this mean the age range is from -20 to 80 years old? A similar issue appears in line 227 “income of 3100.00, (Range +/- 11700)”.

- **Comment 3: Age and income “Range = ±” issue.**

**Response:** Age and income range descriptions have been corrected to accurately reflect minimum and maximum values (line **227-231**).

4. I do not understand the sentences in line 332-333 (“Many are individual focused. However, some will need to be legalized, while others will need to be intervened in.”)

- **Comment 4: Unclear sentence around lines 332–333 (Previous Line Number).**

**Response:** The sentence was written to clearly express the intended meaning. "While numerous approaches prioritize individual agency, it is essential to recognize that certain practices require legal recognition, whereas others necessitate direct intervention." (Line **319-320)**

**Key Components**

1. **Individual Focused:** This suggests that many approaches or solutions concentrate on individual needs, behaviors, or rights. It emphasizes personal responsibility and the role of individual agency in addressing issues.
2. **Legalization:** Some issues or practices may require formal recognition or legalization to ensure they are protected by law. This can involve advocating for rights, changing regulations, or providing legal frameworks that support certain actions or behaviors.
3. **Intervention:** Other issues may necessitate direct intervention, which involves active measures taken to address problems or challenges. This could include social programs, community support, or policy changes aimed at improving conditions or outcomes for individuals or groups.

5. There appears to be a missing right parenthesis in line 445.

**Comment 5: Missing right parenthesis.**

**Response:** Corrected.

**D. Response to Reviewer 2**

**Reviewer #2:** My recommendation is for authors to submit the manuscript for editing as sentences and paragraphs are difficult to follow. Sentences were overly complex, making it difficult to understand and follow a coherent argument/presentation within the manuscript

**General Comment: Manuscript requires language editing; sentences overly complex.**

**Response:** We appreciate the reviewer’s observation regarding sentence complexity. We revised the entire manuscript for clarity, grammar, and coherence. Sentences were restructured to ensure straightforward presentation of ideas. The revised version addresses all issues concerning complex or unclear phrasing.

**Conclusion**

We sincerely thank the editor and reviewers for their valuable feedback. The revisions have substantially improved the quality and clarity of our manuscript. We hope the revised version now meets the standards for publication in PLOS ONE.

We remain available for any further clarification.

**Sincerely,**
**Nakachew Mekonnen Alamirew**

^1^*Department of Health, Behavior, & Society, Faculty of Public Health, Jimma University; Jimma, Oromia, Ethiopia*

^2^*Debre Markos University, Collage of Health Sciences, Department of Public Health, Debre Markos, Ethiopia*

**on behalf of all Co-authors**

*Correspondence author

Email: [nakachewmeku@gmail.com](mailto:nakachewmeku@gmail.com) (NMA)
